# Inhibition of hypoxia-inducible factor via upregulation of von Hippel-Lindau protein induces “angiogenic switch off” in a hepatoma mouse model

**DOI:** 10.1038/mto.2015.20

**Published:** 2015-12-02

**Authors:** Hideki Iwamoto, Toru Nakamura, Hironori Koga, Jesus Izaguirre-Carbonell, Shinji Kamisuki, Fumio Sugawara, Mitsuhiko Abe, Kazuki Iwabata, Yu Ikezono, Takahiko Sakaue, Atsutaka Masuda, Hirohisa Yano, Keisuke Ohta, Masahito Nakano, Shigeo Shimose, Tomotake Shirono, Takuji Torimura

**Affiliations:** 1 Division of Gastroenterology, Department of Medicine, Kurume University School of Medicine, Kurume, Japan; 2 Liver Cancer Division, Research Center for Innovative Cancer Therapy, Kurume University School of Medicine, Kurume, Japan; 3 Department of Applied Biological Science, Faculty of Science and Technology, Tokyo University of Science, Tokyo, Japan; 4 Department of Pathology, Kurume University School of Medicine, Kurume, Japan

## Abstract

“Angiogenic switch off” is one of the ideal therapeutic concepts in the treatment of cancer. However, the specific molecules which can induce “angiogenic switch off” in tumor have not been identified yet. In this study, we focused on von Hippel-Lindau protein (pVHL) in hepatocellular carcinoma (HCC) and investigated the effects of sulfoquinovosyl-acylpropanediol (SQAP), a novel synthetic sulfoglycolipid, for HCC. We examined mutation ratio of *VHL* gene in HCC using 30 HCC samples and we treated the HCC-implanted mice with SQAP. Thirty clinical samples showed no *VHL* genetic mutation in HCC. SQAP significantly inhibited tumor growth by inhibiting angiogenesis in a hepatoma mouse model. SQAP induced tumor “angiogenic switch off” by decreasing hypoxia-inducible factor (HIF)-1, 2α protein via pVHL upregulation. pVHL upregulation decreased HIFα protein levels through different multiple mechanisms: (i) increasing pVHL-dependent HIFα protein degradation; (ii) decreasing HIFα synthesis with decrease of NF-κB expression; and (iii) decrease of tumor hypoxia by vascular normalization. We confirmed these antitumor effects of SQAP by the loss-of-function experiments. We found that SQAP directly bound to and inhibited transglutaminase 2. This study provides evidence that upregulation of tumor pVHL is a promising target, which can induce “angiogenic switch off” in HCC.

## Introduction

Angiogenesis is an essential process for tumor growth and progression.^1^ Newly formed blood vessels in tumor are known to be abnormal and immature structures, which result in high leakiness and less perfusion.^2,3^ Vascular endothelial growth factor (VEGF) family, fibroblast growth factor (FGF) family, angiopoietin, etc. many proangiogenic factors are secreted by not only healthy tissues, but also by cancer cells, which induce neovascularization in tumor.^4,5^ Antiangiogenic therapy has been proposed in 1970s’^1^ and has become one of the standard therapies for several kinds of solid tumors.^6^ Antiangiogenic therapy using sorafenib is the only standard therapy for advanced hepatocellular carcinoma (HCC).^7^ However, the beneficial effects of sorafenib for advanced HCC are limited.^8^ Additionally, many clinical trials using other antiangiogenic drugs were performed for HCC patients. But most of them have failed so far.^9,10^ Therefore, further development of better antiangiogenic therapies are needed to give more benefits for the patients of advanced HCC.

One of the reasons for the modest effects of sorafenib and the other antiangiogenic drugs is acquired resistance for antiangiogenic treatment in tumor. The previously developed antiangiogenic drugs target the specific angiogenic-related molecules or their receptors. Inhibition of the specific molecules results in upregulation of alternative angiogenic factors, so called the “escape phenomenon” in tumor.^11^ For example, inhibition of VEGFR-2 induces alternative upregulation of FGF-2 and angiopoietin-2.^12^ Therapeutic strategy, which targets the specific downstream proteins has limitations in this point. Folkman^13^ who firstly proposed “anti-angiogenic therapy” also proposed the concept of “angiogenic switch” in tumor. This concept is that “switching off” tumor angiogenic potential leads to “tumor dormancy” and is one of the most ideal therapeutic strategies for anticancer treatment. However, the molecules which can induce “angiogenic switch off” in tumor have not been identified yet.

Hypoxic microenvironments are a common feature of solid tumors^14^ and can arise due the proliferative status of cancer cells or an uneven vascular supply in tumor tissues.^15^ Cancer cells adapt to hypoxic environments by activating a number of hypoxia-related pathways, *e.g.*, angiogenic proteins, proliferation, survival, and energy metabolism-related pathways.^14^ Hypoxia-inducible factors 1α and 2α (HIFα proteins) play a central role in these pathways.^16^ HIFα proteins are regulated by prolyl hydroxylase-domain enzyme (PHD) and degraded by Von Hippel-Lindau protein (pVHL) under normoxia. However, hypoxia inhibits activity of PHD and pVHL, resulting in stabilization of HIFα protein.^17^ Therefore, efficient regulation of tumor hypoxia and downregulation of HIFα proteins might be a crucial key for “angiogenic switch off” in tumor. The molecular targeting drug, which targets pVHL has not been reported so much.

**Figure 1:**
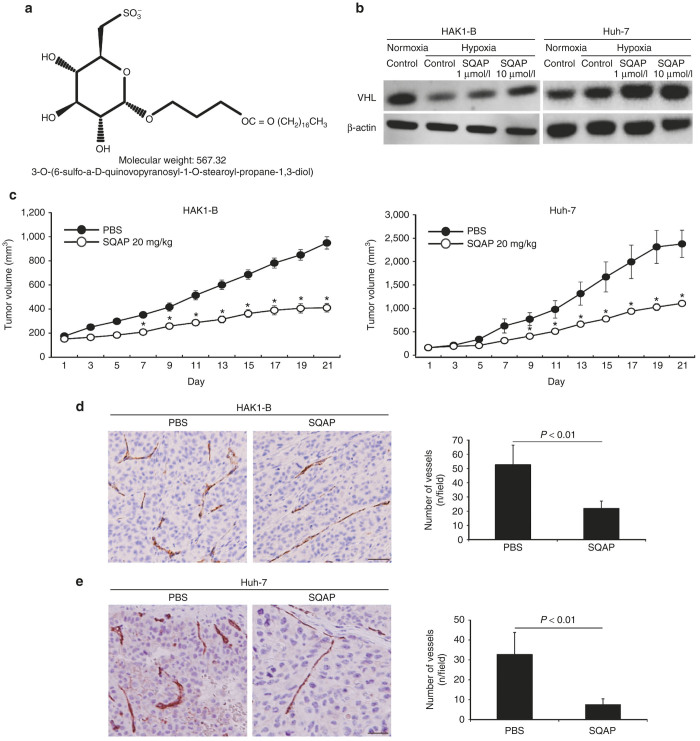
The chemical structure of sulfoquinovosyl-acylpropanediol (SQAP) and the effects of SQAP for HAK1-B and Huh-7 in mice. (**a**) The chemical structure of SQAP. (**b**) SQAP increases the expression of pVHL in HAK1-B and Huh-7. Western blots for both cell lines exposed to SQAP (1 and 10 μmol/l) under hypoxic conditions are shown. Cells were incubated with SQAP-containing medium for 24 hours, after which the cells were moved to 3% O_2_ hypoxic conditions for 24 hours and then lysed with radioimmunoprecipitation buffer. (**c**) Time course of tumor volume changes for subcutaneous tumor-bearing mice treated with SQAP (*n* = 10). HAK-1B and Huh-7 cell line (5 × 10^6^ cells/mouse) were injected into the flank region of the mice. The mice then received the following treatments by intraperitoneal (i.p.) injection: phosphate-buffered saline (control group) or SQAP (20 mg/kg/day, treated group). The treatments were continued for 21 days. *P < 0.05 compared with the control group. All data are represented by mean ± standard deviation (SD). (**d**) SQAP reduced vascularization produced in HAK1-B. (**e**) SQAP reduced vascularization produced in Huh-7. Representative Immunohistochemistry images using CD31 staining in HAK1-B and Huh-7 are shown. The number of tumor vessels are represented as mean ± SD (*n* = 10 per group). Scale bar = 50 μm.

Sulfoquinovosyl-acylglycerols (SQAG) are sulfoglycolipids that were originally derived from sea urchin.^18^ SQAGs can be divided into two groups: monoacyl (SQMG) and diacyl (SQDG), which have one and two fatty acids, respectively. Sahara *et al.* reported that SQMG significantly inhibited tumor growth of breast or lung adenocarcinomas transplanted in nude mice. Mori *et al*.^19^ found that the antitumor effect of SQMG involved antiangiogenesis via downregulation of Tie2 gene expression, but the detailed mechanisms for SQAG action remain unclear. Sulfoquinovosyl-acylpropanediol (SQAP) is SQAG derivative, which is artificially synthesized (Figure 1a).

Here we show that the therapeutic strategy that targets tumor pVHL-HIFα axis is promising for treatment of HCC. Upregulation of pVHL due to SQAP dramatically inhibits HIFα proteins in tumor even under hypoxic condition through multiple mechanisms, which leads “angiogenic switch off” to HCC.

## Results

### Somatic mutation of *VHL* gene is rare in HCC patients

We investigated the mutation rate of the *VHL* gene in 30 HCC patients by DNA sequencing. All evaluated HCC tissues showed a wild-type *VHL* gene profile, regardless of tumor differentiation state (Table 1). This result suggests that *VHL* gene could be a promising therapeutic target for treatment of HCC.

### SQAP upregulates tumor VHL protein in HCC cell lines

We evaluated changes in the amount of pVHL by western blot analysis using cells cultured in SQAP-containing medium. The expression of pVHL was significantly increased by addition of SQAP in a dose-dependent manner (Figure 1b).

### SQAP inhibits tumor growth of HAK1-B and Huh-7 in mice

We examined the antitumor effects and safety of SQAP for HCC-bearing mice. SQAP significantly inhibited tumor growth of HAK1-B and Huh-7 in mice (Figure 1c). With respect to safety, SQAP manifested no overt toxicity in terms of weight loss and myelotoxicity (Supplementary Table S1).

### SQAP induces “angiogenic switch off” in tumor by downregulation of HIFα proteins

To investigate the mechanisms for the antitumor effects of SQAP, we evaluated the degree of tumor cell angiogenesis, apoptosis by immunohistochemistry. Tumor angiogenesis was significantly inhibited and the number of apoptotic cells was significantly increased in both HAK1-B and Huh-7 treated with SQAP (Figure 1d,e and Supplementary Figure S1a,b).

**Figure 2:**
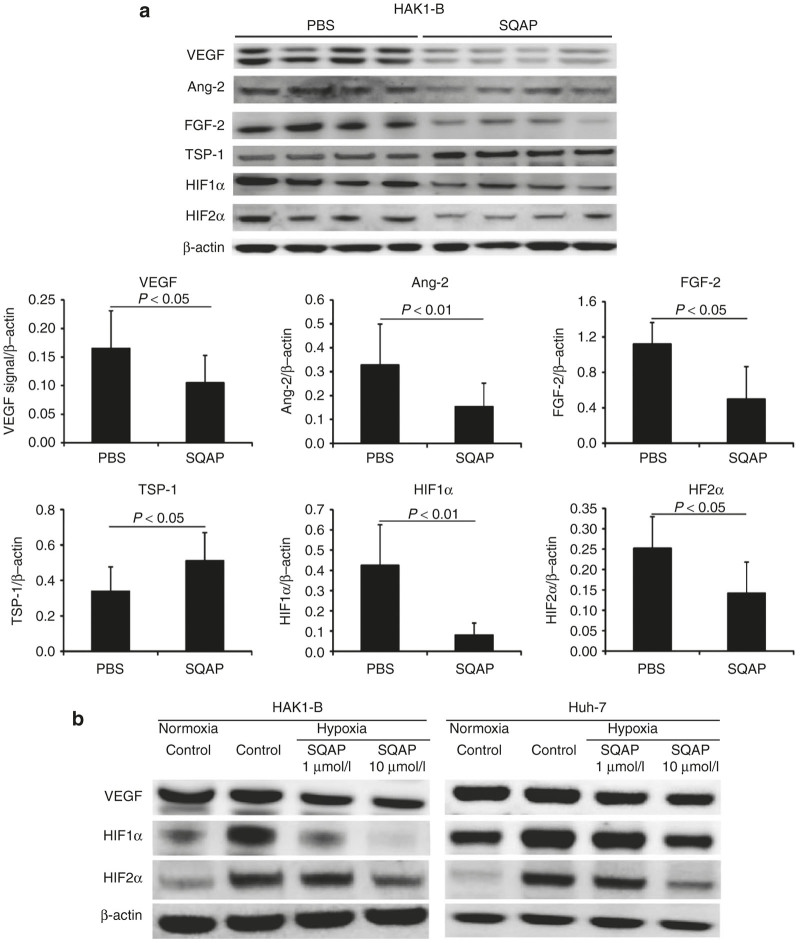
Sulfoquinovosyl-acylpropanediol (SQAP) switches off tumor angiogenic potential by decreasing HIFα protein levels. (**a**) Western blots of vascular endothelial cell growth factor, Ang-2, FGF-2, TSP-1, HIF1α, and HIF2α in HAK1-B tumors treated with SQAP (20 mg/kg/day, i.p injection for 21 days). Whole tumors were collected from tumor-bearing mice (*n* = 4 per group). The band densities for each protein were measured and calibrated by β-actin. All data are represented by mean ± standard deviation. (**b**) Western blots of vascular endothelial cell growth factor, HIF1α, and HIF 2α for HAK1-B and Huh-7 exposed to SQAP under hypoxic conditions. Cells were incubated with SQAP-containing medium for 24 hours, after which the cells were exposed to 3% O_2_ hypoxic conditions for 24 hours and then lysed with radioimmunoprecipitation buffer.

We examined proangiogenic and antiangiogenic proteins in tumor treated with SQAP. The expression of representative proangiogenic proteins (VEGF, FGF-2, and Ang-2) was significantly decreased, while the antiangiogenic protein TSP-1 significantly increased in HAK1-B tumors treated with SQAP (Figure 2a). Noteworthy, HIF1α and HIF2α levels were significantly decreased in HAK1-B tumors treated with SQAP (Figure 2a). And this decrease effect was also shown in Huh-7 tumors treated with SQAP (Supplementary Figure S2).

To confirm whether SQAP could directly affect HIFα proteins expression, we also examined the expression level of HIFα proteins in an *in vitro* assay using HAK1-B and Huh-7 cultured under hypoxia. VEGF expression, the downstream protein of HIF1α was significantly decreased by addition of SQAP in both cell lines (Figure 2b). And the expression of HIF1α and HIF2α was decreased by SQAP in a dose-dependent manner in both cell lines (Figure 2b). These results suggest that SQAP can switch off tumor angiogenic potential by decreasing HIFα protein levels.

### SQAP degrades HIFα proteins through upregulation of pVHL

**Figure 3:**
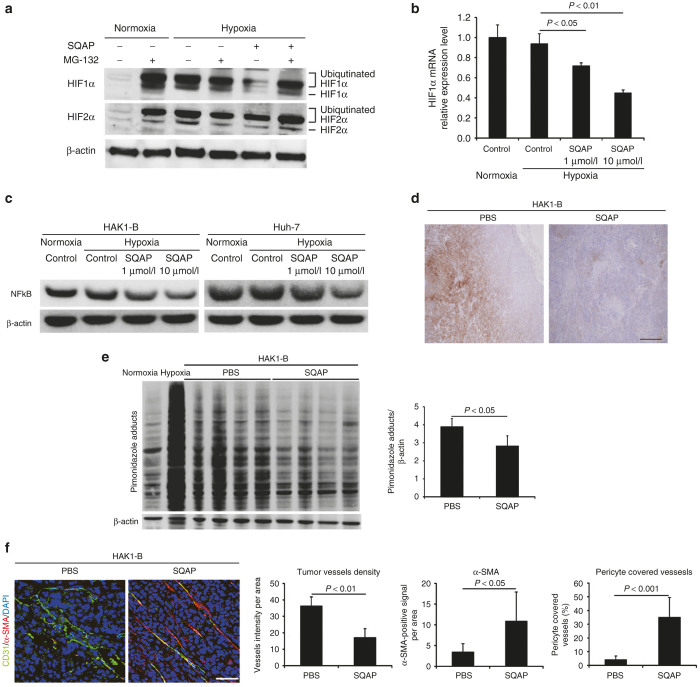
Multiple mechanisms decreasing HIFα in sulfoquinovosyl-acylpropanediol (SQAP). (**a**) The presence of the proteasome inhibitor MG-132 decreased the effect of SQAP on HIFα protein levels. HAK1-B cells incubated under either normoxia or 3% O_2_ hypoxia were treated with 10 μmo/l SQAP for 24 hours and further incubated for 4 hours in the presence of 10 μmo/l MG-132. (**b**) HIF1α synthesis at the transcriptional level decreased upon SQAP addition in a dose-dependent manner. HAK-1B cells were incubated with medium containing different dose of SQAP (1 and 10 μmo/l). Cellular RNA was then extracted and analyzed for HIF1α mRNA expression by quantitative real-time polymerase chain reaction, with GAPDH as a control. (**c**) SQAP decreases NFκB expression in HAK1-B and Huh-7. Western blots for both cell lines exposed to SQAP (1 and 10 μmo/l) under hypoxic conditions are shown. Cells were incubated with SQAP-containing medium for 24 hours, after which the cells were moved to 3% O_2_ hypoxic conditions for 24 hours and then lysed with radioimmunoprecipitation buffer. (**d**) Representative immunohistochemistry images for pimonidazole in HAK1-B tumor treated with SQAP. Scale bar = 200 μm. (**e**) Western blot analysis of pimonidazole adducts in HAK1-B tumors treated with SQAP. Whole tumors were collected from tumor bearing mice (*n* = 4 per group). Negative control: HAK1-B cells cultured under normoxia *in vitro*. Positive control: HAK1-B cell cultured under 3% O_2_ hypoxia *in vitro*. Pimonidazole adducts are shown in the indicated multiple bands. The band densities for each protein were measured and calibrated by β-actin. Average bands densities are represented as mean ± standard deviation (SD). (**f**) Representative double immunofluorescence images using both CD31 (green) and α-SMA (red) in HAK1-B tumors treated with SQAP. Nuclei were stained with DAPI. Scale bar = 50 μm. Relative number of tumor vessels, α-SMA-positive cells, and tumor vessels covered with α-SMA positive cells in HAK1-B tumors treated with SQAP are shown. The number of tumor vessels and the ratio of pericyte covered vessels are represented as mean ± SD. DAPI, 4’,6-diamidino-2-phenylindole, dihydrochloride.

To clarify whether pVHL upregulated by SQAP are involved in decrease of HIFα, we assessed the change of both degradation and synthetic pathways in HIFα proteins. We performed an *in vitro* assay using the proteasomal inhibitor, MG-132 (Z-Leu-Leu-Leu-CHO, Calbiochem, San Diego, CA). In the absence of MG-132, the amount of ubiquitinated HIFα proteins under hypoxic conditions was clearly diminished by SQAP treatment (Figure 3a). However, HIFα protein levels recovered in the presence of MG-132 (Figure 3a). These results suggest that the proteasomal degradation pathway involving pVHL upregulation is responsible for the SQAP-dependent decrease in HIFα protein levels.

### SQAP directly decreases HIFα synthesis and downregulates the expression of NFκB

To ascertain whether SQAP directly decreased HIF1α synthesis at a transcriptional level, we performed quantitative real-time polymerase chain reaction (qRT-PCR) to measure the accumulation of HIF1α mRNA in HAK1-B. qRT-PCR analysis indicated that HIF1α mRNA levels in HAK1-B cells were significantly reduced by the addition of SQAP in a dose-dependent manner (Figure 3b).

To clarify which factors are involved in the reduction of HIFα synthesis, we examined changes of some hypoxia-independent activators for HIFα synthesis using HAK1-B and Huh-7 cultured with SQAP-containing medium under hypoxia. Then we found the presence of SQAP reduced NF-kappaB (NFκB) expression in HAK1-B and Huh-7 cells in a dose-dependent manner (Figure 3c).

### SQAP reduces tumor hypoxia by induction of vascular normalization, contributing to indirect decrease of HIFα proteins

We then performed immunohistochemistry and western blot analysis of HAK1-B tumors added the hypoxia marker pimonidazole to investigate whether SQAP improves tumor hypoxic conditions. The amount of protein bound to pimonidazole was significantly decreased in HAK1-B tissues treated with SQAP, suggesting that tumor hypoxic conditions are improved by treatment with SQAP (Figure 3d,e).

We also performed a double staining assay of α-smooth muscle actin (SMA) and CD31 in HAK1-B tissues. The control group had many tumor vessels with lacked α-SMA-positive cells (Figure 3e). In contrast, tumor vessels were regressed in HAK1-B tumors treated with SQAP. Furthermore, most remaining vessels in treated HAK1-B tumors exhibited α-SMA-covered CD31 vessels, suggesting an increase in the number of normalized vessels (Figure 3f). These results suggest that SQAP improves tumor hypoxia by inducing vascular normalization, which can also contribute to indirect decrease of HIFα proteins.

### Reductions in HIFα and NFκB due to SQAP are restored by *VHL* knockdown

**Figure 4:**
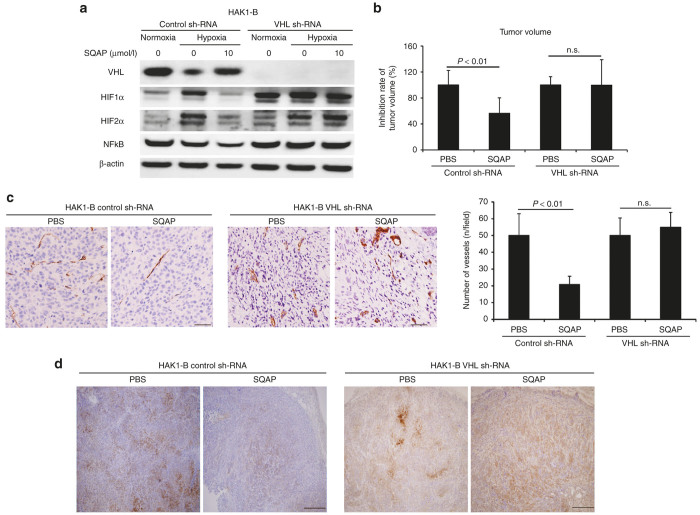
The Effects of sulfoquinovosyl-acylpropanediol (SQAP) in VHL knockdown HAK1-B cell in the *in vitro* and *in vivo* assays. (**a**) Western blots of pVHL, HIF1α, HIF2α, and NFκB in VHL knockdown HAK1-B cell exposed to SQAP under hypoxic condition in an *in vitro* assay. The effect of SQAP on HIF1α, HIF2α, and NFκB protein levels was abolished by *VHL* knockdown. *VHL* knockdown model was made by infecting HAK1-B cells with *VHL* and control sh-RNA lentiviral particles. Cells were incubated with SQAP-containing medium for 24 hours, after which the cells were moved to 3% O_2_ hypoxic conditions for 24 hours and then lysed with radioimmunoprecipitation buffer. (**b**) Decrease rate of tumor volume in SQAP treatment of mice bearing tumors generated by HAK1-B cells expressing either control or *VHL* sh-RNA. Control HAK-1B and VHL knock down HAK-1B (5 × 10^6^ cells/mouse) were injected into the flank region of the mice (*n* = 10). The mice then received the following treatments by intraperitoneal (i.p.) injection: phosphate-buffered saline (PBS) (control group) or SQAP (20 mg/kg/day, treated group). The treatments were continued for 21 days. Tumor volume on sacrifice was measured and the average tumor volume was calculated in each group. Average tumor volume of SQAP treated group in each group, *i.e.*, control and VHL sh-RNA, was divided by that of PBS group in each group. The decrease rate of tumor volume on sacrifice is represented by mean ± standard deviation (SD). (**c**) The effect of SQAP to decrease numbers of vessels in tumors abrogated by downregulation of the *VHL* gene. The number of tumor vessels are represented as mean ± SD (*n* = 10 per group). Scale bar = 50 μm. (**d**) Representative images of pimonidazole in SQAP treatment of mice bearing tumors generated by HAK1-B cells expressing either control or *VHL* sh-RNA. Scale bar = 200 μm.

Upregulation of pVHL by SQAP induced acceleration of HIFα degradation and reduction of HIFα synthesis. To confirm the effects of SQAP, we generated *VHL* knockdown HAK1-B cells. In control sh-RNA HAK1-B cells, SQAP resulted in increased pVHL expression and decreased HIFα and NFκB expression (Figure 4a). In contrast, the effect of SQAP on HIFα and NFκB protein levels was absent in *VHL* knockdown HAK1-B cells (Figure 4a). This result suggests that upregulation of VHL by SQAP is essential for the decrease of HIFα expression.

### SQAP did not inhibit tumor growth, tumor angiogenesis in VHL knockdown HAK1-B in mice

We also performed *in vivo* assays using *VHL* knockdown HAK1-B cells. SQAP inhibited tumor growth, tumor angiogenesis, and improved tumor hypoxia in control sh-RNA HAK1-B tumors. However, SQAP could not inhibit tumor growth, tumor angiogenesis and reduce tumor hypoxia in VHL knockdown HAK1-B cells (Figure 4b–d).

### SQAP did not show any *in vivo* effects and *in vitro* effects for KYN-2 cell line, *VHL* naturally mutated HCC cell line

**Figure 5:**
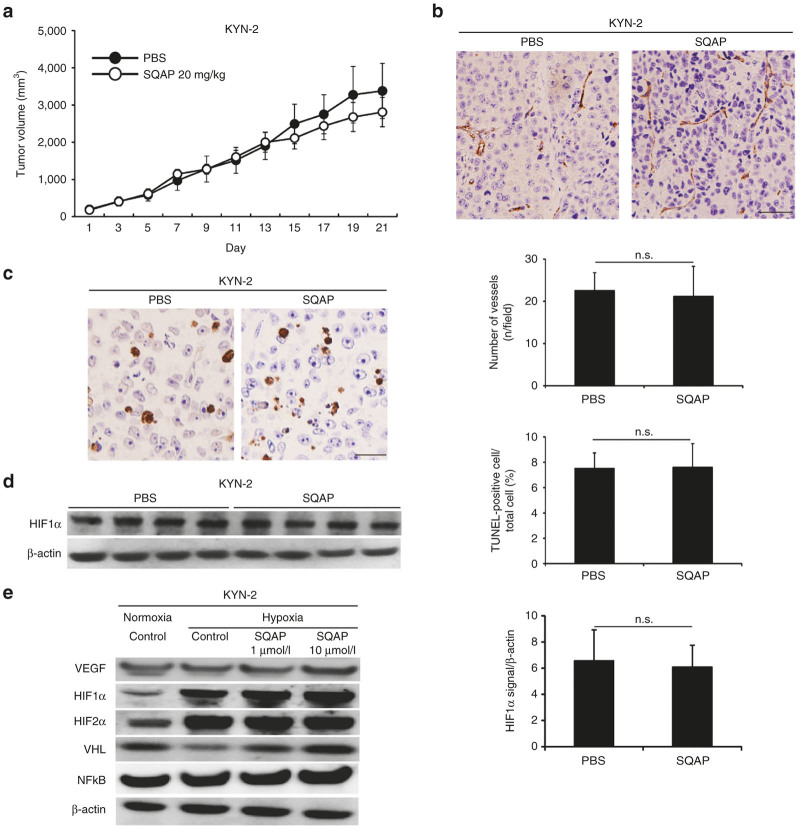
The effects of sulfoquinovosyl-acylpropanediol (SQAP) for KYN-2, *VHL* naturally mutated HCC cell line. (**a**) Time course of tumor volume changes for subcutaneous KYN-2-bearing mice treated with SQAP (*n* = 10 per group). All data are represented by mean ± standard deviation (SD). The mice then received the following treatments by intraperitoneal (i.p.) injection: phosphate-buffered saline (control group) or SQAP (20 mg/kg/day, treated group). The treatments were continued for 21 days. (**b**) Representative Immunohistochemistry images of CD31 staining in KYN-2 are shown. The number of tumor vessels are represented as mean ± SD (*n* = 10 per group). Scale bar = 50 μm. (**c**) Representative immunohistochemistry images of TUNEL staining in KYN-2 are shown. The number of apoptotic cells is represented as mean ± SD. (**d**) Western blots of HIF1α in KYN-2 tumors treated with SQAP. Whole tumors were collected from tumor bearing mice (*n* = 4 per group). The band densities for each protein were measured and normalized by β-actin. The average band densities are represented as mean ± SD. (**e**) Western blots of vascular endothelial cell growth factor, HIF1α, HIF2α, VHL, and NFκB for KYN-2 exposed to SQAP under hypoxic conditions are shown. Cells were incubated with SQAP-containing medium for 24 hours, after which the cells were moved to 3% O_2_ hypoxic conditions for 24 hours and then lysed with radioimmunoprecipitation buffer. TUNEL, TdT-mediated dUTP nick end labeling.

Since artificial ablation of *VHL* gene caused complete loss of effects in SQAP, next we evaluated the effects of SQAP in *VHL* naturally mutated HCC cell line. We performed *VHL* genetic mutation analysis using direct DNA sequencing. Then we found KYN-2 had a point mutation of *VHL* gene, while SQAP-sensitive cell lines both HAK1-B and Huh-7 cells were wild-type *VHL* gene (Supplementary Table S2). Although there was no significant difference in IC_50_ value of SQAP among KYN-2, HAK1-B, and Huh-7 (Supplementary Table S3), SQAP could not inhibit tumor growth of KYN-2 in mice (Figure 5a). Both SQAP did not inhibit tumor angiogenesis and did not increase apoptosis in KYN-2 tumors (Figure 5b,c). SQAP did not decrease the expression of HIF1α in KYN-2 tumors (Figure 5d). Also in an *in vitro* assay, VEGF, HIFα proteins, and NFκB expression did not decrease by addition of SQAP, in spite of increasing pVHL expression (Figure 5e). Loss-of-function experiments using artificial ablation of VHL gene and the experiments using VHL naturally mutated HCC cell line clearly supported that pVHL is essential for antitumor effects of SQAP.

### SQAP directly binds with TG2 and inhibits TG2 activity in a dose-dependent manner

**Figure 6:**
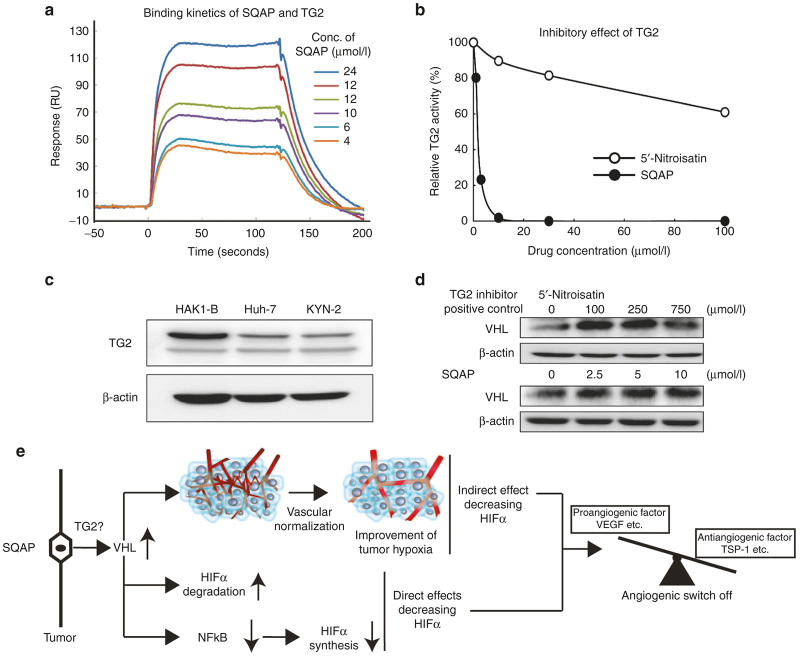
Sulfoquinovosyl-acylpropanediol (SQAP) directly binds with and inhibits TG2 activity. (**a**) Binding analysis of TG2 with SQAP. An SPR analysis for the binding between SQAP and human TG2 was performed. SQAP was injected over flow cells on the immobilized TG2. The background resulting from the injection of running buffer was subtracted from the data before plotting. Resonance units (RU) were generated by subtraction of the background signal generated simultaneously on the control flow cell. (**b**) Inhibition of tissue transglutaminase activity. SQAP (filled circle) strongly inhibited the activity of TG2 compared to 5’-Nitroisatin (open circle). The horizontal axis indicates the concentration of drugs, and the vertical axis indicates the relative activity. (**c**) Western blots of TG2 in HAK1-B, Huh-7 and KYN-2. All HCC cell line we used expresses TG2. Cells were incubated under normoxia for 24 hours and then lysed with radioimmunoprecipitation buffer. (**d**) Western blots of pVHL in HAK1-B exposed different concentration of 5’-Nitroisatin and SQAP. 5’-Nitroisatin was used as a positive control for TG2 inhibitor. HAK1-B was incubated with different dose of 5’-Nitroisatin (100, 250, and 750 μmol/l) and SQAP (2.5, 5.0, and 10 μmol/l) under normoxia for 24 hours and then lysed with radioimmunoprecipitation buffer. (**e**) Summary of the multiple mechanisms decreasing HIFα of SQAP. SQAP upregulates tumor pVHL. Upregulated pVHL accelerates HIFα degradation. Moreover, upregulated pVHL decreases NFκB expression, resulting in decrease of HIFα synthesis. In addition to these direct effects decreasing HIFα, upregulated pVHL improves tumor hypoxia by induction of vascular normalization, which contributes to indirect decrease of HIFα proteins. All mechanisms contribute to induction of “angiogenic switch off” in tumor. SQAP directly binds and inhibits tumor TG2, implying that SQAP upregulates pVHL via inhibition of TG2.

One remaining question was that how SQAP upregulates the expression of pVHL in cancer cells. Kim *et al*.^20^ recently reported that genetic ablation of TG2 resulted in upregulation of pVHL. Therefore, we firstly performed the binding assay of SQAP and TG2. Then we found that SQAP directly bound with TG2 in a dose-dependent manner (Figure 6a). Additionally, in TG2 activity assay, SQAP showed a stronger inhibitory effect of TG2 activity in a dose-dependent manner, compared with 5’-Nitroisatin, a TG2 inhibitor as a positive control (Figure 6b). Next, we examined whether HCC cell lines, which we used expressed TG2. HAK1-B, Huh-7, and KYN-2 expressed TG2 in western blot analysis (Figure 6c). 5’-Nitroisatin upregulated pVHL expression in HAK1-B cell in a dose-dependent manner (Figure 6d). Similarly, SQAP upregulated pVHL expression of HAK1-B in a dose-dependent manner (Figure 6d).

## Discussion

Mutations of the *VHL* gene are often involved in tumorigenesis and cancer progression, which is supported by the finding that the *VHL* gene shows somatic mutations in more than 70% of clear-cell renal carcinomas.^21^ However, as shown in Table 1 and Piao *et al*.^22^, *VHL* mutations in HCC are very rare. Therefore, VHL can be a promising target for molecular targeting therapy of HCC.

In this study, we demonstrated that SQAP inhibited tumor angiogenesis by upregulation of pVHL in tumors, which resulted in a significant antitumor effect for HCC in mice without overt toxicity. SQAP decreased proangiogenic factors and increased endogenous angiognenic inhibitor through downregulation of HIFα proteins, which finally reduced proangiogenic potential of HCC cells. This phenomenon is called “angiogenic switch off”. Upregulating pVHL by SQAP showed multiple mechanisms that decrease the expression of HIFα proteins. Firstly, upregulation of pVHL accelerated HIFα degradation as a direct mechanism. Second, upregulation of pVHL downregulated NFκB expression, which reduced HIF1α synthesis at a transcriptional level. Finally, upregulation of pVHL improved tumor hypoxic microenvironment by induction of vascular normalization (Figure 6e). All these effects were abolished by *VHL* knockdown or *VHL* genetic mutation, indicating that pVHL upregulation is a critical factor for the effects of SQAP.

Overexpression of HIFα proteins has been shown in HCC.^23^ HIFα proteins regulate the expression of many angiogenic factors, which makes HIFα a central player in the “angiogenic switch”.^24^ In the present *in vivo* study, HIFα protein downregulation in SQAP switched off the angiogenic potential of tumors. Many antiangiogenic agents such as sorafenib or bevacizumab produce antitumor effects by inhibiting specific molecular targets in endothelial or cancer cells.^8^ The inhibition of specific molecular targets can induce upregulation of other growth factors, inducing the “escape phenomenon”.^11^ This alternative upregulated pathways result in drug resistance of HCC, which causes less clinical benefit. Inhibition of HIFα proteins can downregulate multiple angiogenic factors expressed specifically by cancer cells. Therefore, HIFα inhibitors can be promising drugs, which induce the “angiogenic switch off.” 

HIFα inhibitors can be divided into two groups based on their inhibitory mechanisms, with one group downregulating HIFα synthetic pathways that involve in inhibition of PI3K, RAS, or elf2α and the other promoting HIFα degradation by enhancing the activity of HIF-degrading pathways.^25,26^ SQAP is a unique agent that has qualities of both types of HIFα inhibitors via its upregulation of pVHL. VHL is a central player in HIFα protein degradation and SQAP could indeed decrease HIFα proteins levels by activating the VHL-proteasome-dependent degradation pathway. Additionally, pVHL upregulation was also able to downregulate NFκB. Rius *et al*.^27^ have reported that NFκB regulated *HIFα* expression at the transcriptional level in hypoxia-independent manner. And NFκB in turn is negatively regulated by pVHL.^28^ In this study, upregulation of pVHL decreased NFκB expression and genetic ablation of *VHL* showed restoration of NFκB expression, suggesting that pVHL directly regulates the expression of NFκB. The results shown in the present study support these findings, indicating that HIFα proteins can be regulated by pVHL not only through HIFα degradation, but also by affecting HIFα synthesis. SQAP also possessed an indirect effect whereby HIFα protein levels decrease in response to improved tumor hypoxia caused by vascular normalization. There have been no reports describing the role for pVHL in inducing vascular normalization, although the results presented by Ohta *et al*.^29^ do support this evidence in that the SQAP analog SQMG also showed vascular normalization in response to a combination of low-dose SQMG treatment and radiation therapy. Improvement of tumor hypoxia by SQAP also supports acceleration of HIFα proteins degradation by pVHL, indirectly. HIFα proteins are degraded by binding with PHDs and pVHL.^17^ During hypoxia, PHD activity is inhibited, which leads to increase in the amount of stabilized HIFα proteins. Therefore, improvement of tumor hypoxia by SQAP could indirectly increase degradation of HIFα proteins. Taken together, these findings indicate that pVHL decreases HIFα protein levels by multiple mechanisms, suggesting that pVHL upregulation by SQAP could be the promising target, which can induce tumor “angiogenic switch off.” 

In this study, we showed that SQAP directly binds with TG2 and strongly inhibits TG2 activity. Kim *et al*.^20^ reported that TG2 can polymerize VHL, which results directly in the depletion of VHL through ubiquitination and proteasomal degradation. They support our findings that genetic ablation of TG2 resulted in upregulation of pVHL. These findings imply that we might be able to regulate “angiogenic switch” from tumor cell surface through inhibition of TG2 activity. We need further investigation to know about the role of TG2 inhibition in induction of tumor “angiogenic switch off.” 

In summary, we showed that tumor pVHL can be a promising target to induce “angiogenic switch off” in HCC and have demonstrated that SQAP can efficiently upregulates pVHL in HCC, which resulted in significant inhibition of tumor growth with inhibiting tumor angiogenesis. These effects were caused by downregulation of HIFα proteins through multiple direct and indirect mechanisms. SQAP could be a next-generation antiangiogenic drug in the treatment of HCC.

## Materials and Methods

### Cell lines and culture

The human HCC cells HAK1-B and KYN-2 were provided by the Department of Pathology, Kurume University School of Medicine, while Huh-7 cells were obtained from Cambrex (Walkersville, MD). Cells were maintained in Dulbecco modified Eagle medium (Gibco Invitrogen Cell Culture, Auckland, New Zealand) supplemented with 10% fetal bovine serum (Biowest, Nuaille, France). Human umbilical vascular endothelial cells were purchased from Cambrex and maintained in endothelial cell growth medium-2 (Clonetics, San Diego, CA) containing 10% fetal bovine serum. All cell lines were maintained at 37 °C in a humidified atmosphere with 5% CO_2_. For *in vitro* hypoxic assays, cells were cultivated in hypoxic conditions that were maintained by a hypoxia chamber (APM-30D, ASTEC, Fukuoka, Japan) flushed with a gas mixture of 3% O_2_, 5% CO_2_, and 92% N_2_.

### Drugs

SQAP (Figure 1a) was provided from CANGO (Tokyo, Japan) and dissolved in DMSO. Stock solutions were appropriately diluted with phosphate-buffered saline so that the final DMSO concentration was less than 0.5%.

### *In vivo* xenograft assay

Male 5-week-old nude mice (BALB/c nu/nu) were purchased from Kyudo KK (Fukuoka, Japan) and housed in specific pathogen-free conditions. All animal studies were approved by the Kurume University Animal Care and Use Committee. HAK1-B, Huh-7, and KYN-2 cells (5 × 10^6^ cells/mouse) suspended in phosphate-buffered saline were injected subcutaneously into the mouse flank region. The tumor volume (*V* = mm^3^) and mouse body weight were measured every 2 days and estimated using the following equation: *V* = 0.5 × length × width^2^. When the estimated tumor volume reached 150–200 mm^3^, the mice received intraperitoneal (i.p.) injections of either phosphate-buffered saline (control group) or SQAP (20 mg/kg/day, treated group). The number of mice in each group was 10. Treatments were continued for 21 days and on day 22, tumor tissue and blood samples were collected after anesthesia using nembutal (0.1 ml/g body weight) by i.p. injection. Myelotoxicity was evaluated using the blood samples.

### Western blot analysis

Cells were lysed with radioimmunoprecipitation buffer containing 10 mmol/l NaF, 1 mmol/l Na_3_VO_4_, 10 mmol/l dithiothreitol, 1 mmol/l phenylmethylsulfonyl fluoride, and a phosphatase inhibitor (Thermo Scientific, Rockford, IL). Western blot analyses were performed as previously described.^30^ For *in vitro* hypoxic assays using western blot analysis, cells were incubated with medium containing SQAP (1 and 10 μmol/l) for 24 hours whereupon cells were moved to the hypoxic chamber with 3% O_2_ for 24 hours and then lysed with radioimmunoprecipitation buffer. Rabbit primary antibodies and dilutions used were: anti-extracellular signal-regulated kinase 1/2 (ERK1/2) antibody (1:200), anti-phosphorylated ERK1/2 antibody (1:200), anti-mammalian target of rapamycin antibody (1:200), anti-phosphorylated mammalian target of rapamycin antibody (1:200), anti-VHL antibody (1:300), and anti-NFκB antibody (1:250). Rabbit anti-angiopoietin-2 (Ang-2) and FGF-2 antibodies were from Santa Cruz Biotechnology (Santa Cruz, CA) and used at 1:100 dilution. Rabbit anti-Thrombospondin-1 (TSP-1), anti-VEGF, and anti-HIF2α antibodies were from Abcam (Tokyo, Japan) and used at dilutions of 1:300 and 1:250, and 1:250, respectively. Mouse anti-HIF1α antibody (1:500) was from BD Biosciences (Tokyo, Japan). Rabbit Anti-TG2 antibody was from Millipore (1:500; Merck Millipore, MA). Mouse Anti-β-actin antibody (1:1,000; Sigma, St Louis, MO) was used as an internal loading control. Visualization of the protein signal was achieved with horseradish peroxidase–conjugated secondary antibodies (1:5,000; GE Healthcare UK, Buckinghamshire, UK) enhanced by chemiluminescence in western blot analysis system (Amarsham Pharmacia Biotech, Piscataway, NJ) using LAS 4000 mini (Fujifilm, Tokyo, Japan). The amount of luminescence in each sample was quantified by multigauge software (Fujifilm).

### Immunohistochemistry

Immunohistochemical staining of CD31 and TdT-mediated dUTP nick end labeling staining was performed as previously described.^5,31^ For double immunofluorescence examination of CD31 and α-smooth muscle actin (α-SMA), sections were incubated at 4 °C overnight with rabbit anti-human CD31 antibody (Abcam) and fluorescein isothiocyanate–conjugated anti-rabbit IgG antibody (1:100; Invitrogen, Carlsbad, CA) for 1 hour. After blocking, sections were incubated with goat anti-human α-SMA antibody (1:100; Abcam) at 4 °C overnight and Alexa fluor 568-labeled anti-goat IgG antibody (1:100; Invitrogen) for 1 hour. Nuclei were counterstained with TO-PRO-3 iodide (1:1,000, Invitrogen). Imaging was performed in a six-border zone region for each sample (z-series Zeiss LSM-510 Meta Confocal Microscope, Carl Zeiss, Jena, Germany) and analyzed with Zeiss LSM Image Browser software version 3.5 (Carl Zeiss).

### RNA extraction and qRT-PCR assay

Total RNA was extracted using the RNA assay kit (RNeasy mini kit, QIAGEN, Tokyo, Japan) according to the manufacturer’s instructions. qRT-PCR analysis was performed using power SYBR Green PCR Master Mix (Applied Biosystems, Warrington, UK). The primer sequences were as follows: HIF1α, forward, 5′-TGCTCATCAGTTGCCACTTCC-3′, and reverse, 5′-CCAAATCACCAGCATCCAGAAGT-3′; and GAPDH forward, 5′-CATGAGAAGTATGACAACAGCC-3′, and reverse, 5′-AGTCCTTCCACGATACCAAAG-3′. The housekeeping gene *GAPDH* was used as a standard. qRT-PCR was performed using the ABI PRISM 7000 Sequence Detection System (Applied Biosystems) according to the manufacturer’s instructions.

### *In vivo* hypoxic assays using pimonidazole

To assess *in vivo* hypoxic conditions in tumors, pimonidazole (60 mg/kg/body weight; Hypoxyprobe-1, NPI, Belmont, MA) was intraperitoneally injected into mice bearing SQAP-treated (14 days) HAK1-B-generated tumors (*n* = 4). Two hours after injection, tumor tissues were collected under anesthesia. Immunohistochemistry using pimonidazole was performed according to the manufacturer’s instructions. Pimonidazole adduct formation was evaluated by western blotting.^4,32^ Pimonidazole adducts were shown as the multiple bands, reflecting its capacity to bind to a variety of proteins under hypoxic conditions. The western blot analysis was performed using LAS 4000 mini (Fujifilm) and the protein amounts in the bands for each sample were evaluated using multigauge software (Fujifilm).

### *VHL* sh-RNA knockdown assay

All experiments regarding genetic transformation were approved by the Kurume University Genetic Modifications Safety Committee. VHL sh-RNA knockdown assays were performed as previously described.^5,33^ VHL and control sh-RNA lentiviral particles were purchased from Santa Cruz Biotechnology and HAK1-B cells were infected with these lentiviral particles and generated the stable VHL knockdown and control cells according to the manufacturer’s instructions.

### DNA extraction and *VHL* mutation analysis

The human *VHL* gene is located on the short arm of chromosome 3 and contains 3 exons. Genomic DNA was extracted from each HCC cell line and primary tissues from 30 HCC patients using a Nucleospin Tissue kit (Macherey-Nagel, Duren, Germany) and subsequently PCR-amplified. Human primary HCC samples were collected when hepatectomy was performed and then freezed in liquid nitrogen. Informed consent was obtained from all patients and the *VHL* mutation analyses were approved by the ethical committee of Kurume University. PCR products were assessed by DNA sequencing performed by the Dragon Genomics Center, Takara Bio (Otsu, Japan).

### Surface plasmon resonance

The binding kinetics of SQAP and human TG2 were analyzed by surface plasmon resonance (SPR) biosensor (Biacore 3000, GE Healthcare, Tokyo, Japan). The surface of a CM5 sensor chip was activated by injecting a solution containing 37.5 mg/ml 1-ethyl-3-(3-dimethylaminopropyl) carbodiimide hydrochloride and 5.8 mg/ml N-hydroxysuccinimide at 10 µl/minute rate for 7 minutes. Recombinant TG2 (Immundiagnostik AG) was injected over the sensor chip and captured on the carboxymethyl dextran matrix via amine coupling reaction. The surface was then blocked by injecting 1 M ethanolamine hydrochloride at pH 8.5 for 7 minutes. Binding analysis was performed in HBS-P (10 mmol/l Hepes, 0.15M NaCl, 0.005% Surfactant P20, pH 7.4) buffer using a flow rate of 20 µl/minute at 25 °C. Various concentrations of SQAP were successively injected upon the immobilized TG2 to detect the SPR response generated. BIAevaluation 4.1 software (GE Healthcare) was used to determine the kinetic parameters.

### Tissue transglutaminase assay

Transglutaminase assay was performed using Transglutaminase Assay Kit (Sigma-Aldrich, Tokyo, Japan), followed the instruction. Briefly, 50 μl of SQAP aqueous solution was added to each well at different concentrations, and the same volume of assay mixture contained in the kit was also added. After incubation at room temperature for 30 minutes, each well was washed with ddH_2_O three times, and then 100 μl of streptavidin peroxidase solution was added to each well and incubated at room temperature for 20 minutes. Each well was added 180 μl of 3,3’,5,5’-tetramethylbenzidine liquid substrate system. Reaction was stopped with addition of stop solution as soon as the color developed, and was measured the absorption at 450 nm. 5’-Nitroisatin (Sigma-Aldrich) was used as a positive control.^34^

### Statistical analysis

All experimental data are expressed as the mean ± standard deviation. Differences between groups were examined for statistical significance using the Mann–Whitney *U*-test and nonparametric analysis of variance. If the one-way analysis of variance was significant, differences between the individual groups were estimated using Fisher’s least significant difference test. Differences with *P* < 0.05 were considered statistically significant.

## Figures and Tables

**Table 1:** Genetic analysis of the *VHL* gene in 30 HCC patients

	*Thirty HCC patients*
Differentiation^a^ (well/mode/poor)	8/22/0
VHL somatic mutation^b^ (Wt/Mt)	0/30

HCC, hepatocellular carcinoma; VHL; von Hippel-Lindau.

a well = well, mode = moderately, poor = poorly.

b Wt = wild type, Mt = mutation.
