# Supplementary figures and images for: Inhibition of hypoxia-inducible factor via upregulation of von Hippel-Lindau protein induces “angiogenic switch off” in a hepatoma mouse model
Source: Mol Ther Oncolytics. 2015 Dec 2;2:15020–. doi: 10.1038/mto.2015.20 (PMC4782957; doi:10.1038/mto.2015.20)

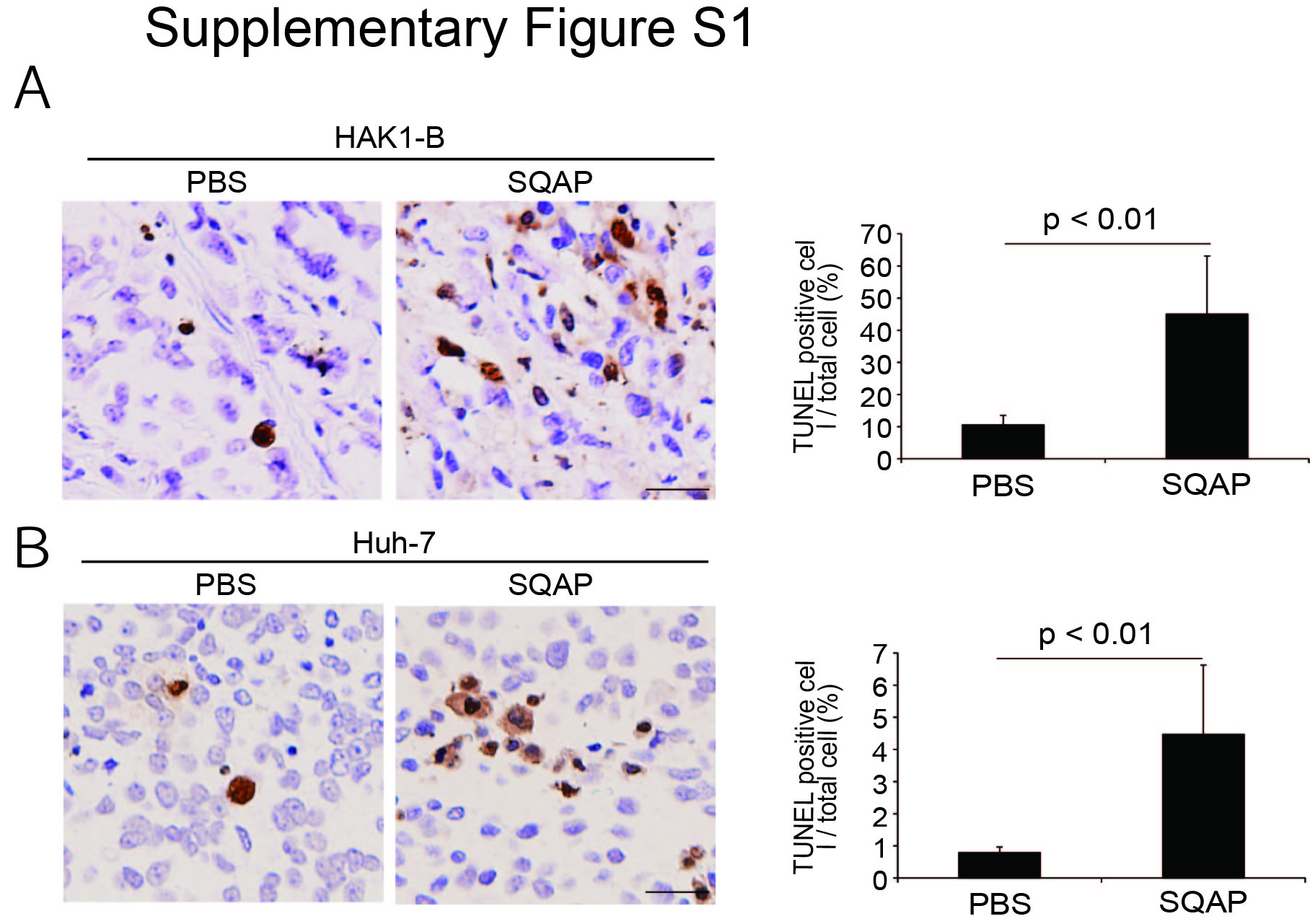

Supplement: Supplementary Figure S1 [file mto201520-s1.jpg]

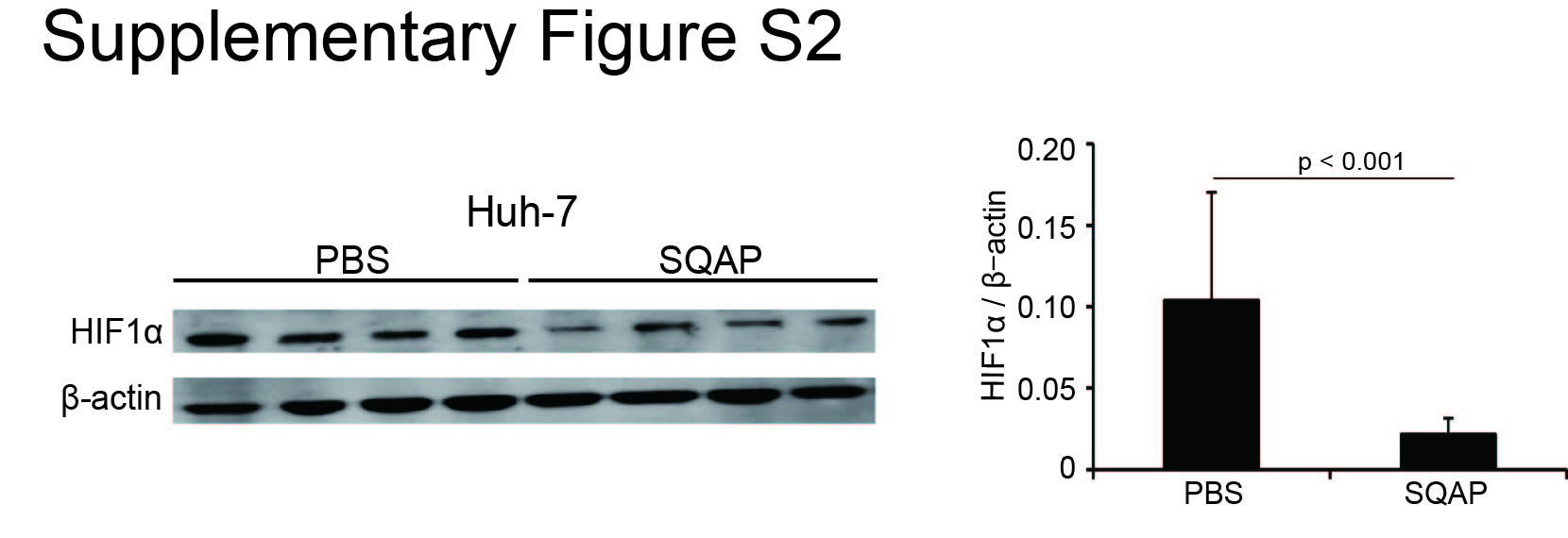

Supplement: Supplementary Figure S2 [file mto201520-s2.jpg]
