# Supplementary material for: Inhibition of hypoxia-inducible factor via upregulation of von Hippel-Lindau protein induces “angiogenic switch off” in a hepatoma mouse model
Source: Mol Ther Oncolytics. 2015 Dec 2;2:15020–. doi: 10.1038/mto.2015.20 (PMC4782957; doi:10.1038/mto.2015.20)
Supplement: Supplementary Tables and Figure Legends [file mto201520-s3.docx]

**Supplementary Informations**

Table S1

|  | HAK1-B | | Huh-7 | | KYN-2 | |
| --- | --- | --- | --- | --- | --- | --- |
|  | PBS | SQAP | PBS | SQAP | PBS | SQAP |
| Body weight change (%) | 112.1±4.1 | 106.3±6.6 | 100.2±7.0 | 107.4±10.2 | 103.0±11.2 | 97.3±9.7 |
| Hemoglobin level (mg/dl) | 16.3±0.2 | 14.8±2.1 | 14.2±1.9 | 17.1±1.3 | 13.6±1.1 | 13.4±2.8 |
| Leukocyte count (×10^3^/μl) | 4.2±4.1 | 4.9±3.7 | 6.7±0.3 | 6.3±0.5 | 3.9±2.3 | 4.0±1.3 |
| Platelet count (×10^4^/μl) | 66.8±16.7 | 66.6±14.0 | 76.4±10.6 | 78.9±6.3 | 94.7±16.7 | 132.3±23.2 |

**Assessment of SQAP toxicity in tumor-bearing mice**

There were no significant differences in all toxicity profiles between the control and SQAP treatment groups. Body weight change (%) was calculated by: (body weight on final treatment day) / (body weight on initial treatment day) ×100. After treatment with SQAP for 21 days, mouse blood was collected under anesthesia. All data are represented by mean ±SD.

**Genetic analysis of the *VHL* gene in HCC cell lines**

Table S2

| Cell line | Location (exon) | Position | Nucleotide change | Zygosity |
| --- | --- | --- | --- | --- |
| HAK1-B |  |  |  | wild type |
| Huh-7 |  |  |  | wild type |
| **KYN-2** | **2** | **10188316** | **G to A** | **heterozygote** |

Table S3

| **Effect of SQAP on the viability of tumor *in vitro*** | |
| --- | --- |
| HCC cell line | SQAP IC_50_ (μM) |
| HAK1-B | 10.41±0.12 |
| Huh-7 | 10.31±0.08 |
| KYN-2  HCC; hepatocellular carcinoma | 10.59±0.04 |

**Figure S1. SQAP induces apoptosis for HAK1-B and Huh-7 tissues**

a and b SQAP treatment increased the number of apoptotic cells in HAK1-B and Huh-7 tissues. Representative TUNEL staining images of HAK1-B and Huh-7 are shown. The apoptotic rate was calculated by: (number of TUNEL-positive cells)/ (all cells in a tumor field) and is represented as mean ± SD (n = 10 per group). Scale bar = 100 μm

**Figure S2. SQAP downregulates HIF1α expression in Huh-7 tissue**

Western blots of HIF1α in Huh-7 tissues treated with SQAP. The band densities for each protein were measured and normalized by β-actin.
